# Compound heterozygous variants in the *ABCG8* gene in a Japanese girl with sitosterolemia

**DOI:** 10.1038/s41439-020-00112-y

**Published:** 2020-09-14

**Authors:** Nobuhiro Hashimoto, Sumito Dateki, Eri Suzuki, Takatoshi Tsuchihashi, Aiko Isobe, Sari Banno, Tomoka Kageyama, Naonori Maeda, Naomi Hatabu, Rieko Sato, Masashi Miharu, Hisayo Fujita, Osamu Komiyama, Hitomi Shimizu, Tomonobu Hasegawa, Kazuki Yamazawa

**Affiliations:** 1 Department of Pediatrics, National Hospital Organization Tokyo Medical Center, Tokyo, Japan; 2 Department of Pediatrics, Nagasaki University Graduate School of Biomedical Sciences, Nagasaki, Japan; 3 Department of Pediatrics, Kawasaki Municipal Hospital, Kanagawa, Japan; 4 Department of Pediatrics, Keio University School of Medicine, Tokyo, Japan; 5 Medical Genetics Center, National Hospital Organization Tokyo Medical Center, Tokyo, Japan

**Keywords:** Disease genetics, Dyslipidaemias

## Abstract

Sitosterolemia is an autosomal recessive disorder that affects lipid metabolism and is characterized by elevated serum plant sterol levels, xanthomas, and accelerated atherosclerosis. In this study, we report a novel nonsense single-nucleotide variant, c.225G > A (p.Trp75*), and an East Asian population-specific missense multiple-nucleotide variant, c.1256_1257delTCinsAA (p.Ile419Lys), in the *ABCG8* gene in a compound heterozygous state observed in a Japanese girl with sitosterolemia.

Sitosterolemia (OMIM #210250) is a rare autosomal recessive disorder characterized by elevated serum plant sterol levels, xanthomas, and accelerated atherosclerosis^1^. Biallelic pathogenic variants of the *ABCG5* and *ABCG8* genes, encoding the sterol efflux transporter ABCG5 (sterolin-1) and ABCG8 (sterolin-2), respectively, cause increased intestinal absorption and decreased biliary excretion of plant sterols, leading to sitosterolemia^2,3^. Here, we report a case of sitosterolemia caused by a novel nonsense variant and an East Asian population-specific missense variant in the *ABCG8* gene in a compound heterozygous state.

The proband is a Japanese girl born as the third child to nonconsanguineous parents. Family history did not reveal any remarkable genetic disorders. The proband developed xanthomas on her wrists when she was 6 months old, which worsened with age. She was referred to our hospital for polyarthralgia at the age of 6 years. Physical examinations revealed multiple xanthomas on her wrists, knees, Achilles tendons, and hip region (Fig. 1a–c). Atherosclerotic plaque in the left carotid artery and aortic valve regurgitation were detected by ultrasound examinations. Blood tests showed an elevated serum total cholesterol level of 423 mg/dL and low-density lipoprotein-cholesterol level of 370 mg/dL. High-density lipoprotein-cholesterol and triglyceride levels were normal. Further investigations revealed that her serum plant sterol levels were markedly elevated: the sitosterol level was 223.5 µg/mL (reference: 2.4 ± 0.73 µg/mL), campesterol level was 109.2 µg/mL (reference: 4.89 ± 1.37 µg/mL), and stigmasterol level was 6.6 µg/mL (reference: 2.57 ± 1.37 µg/mL). Her parents and sisters showed normal serum lipid profiles.

**Fig. 1:**
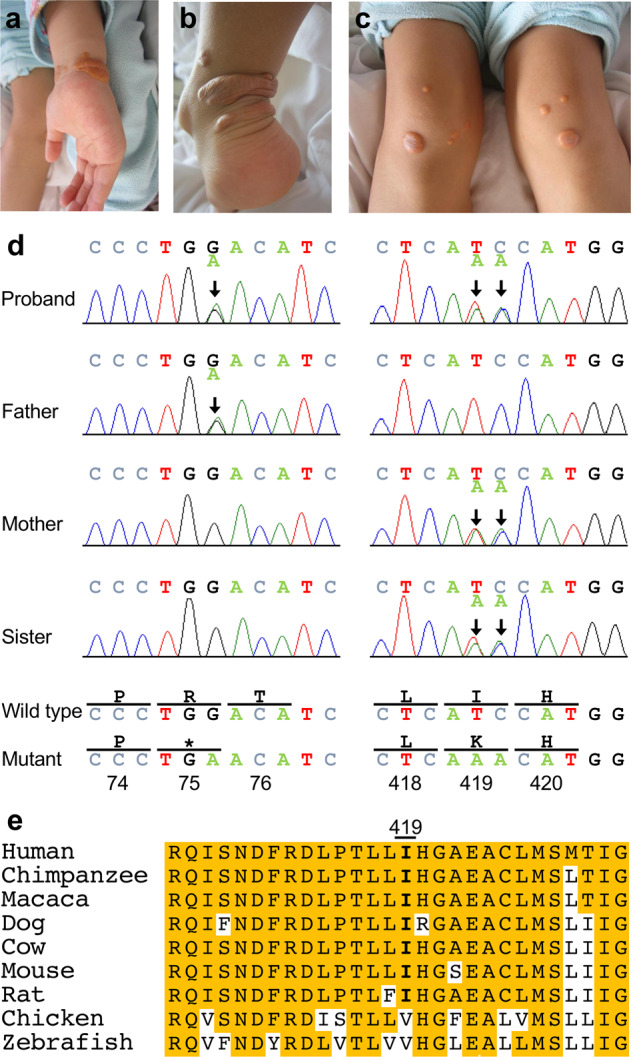
Clinical and genetic features of the proband. Xanthomas seen on the **a** wrist, **b** Achilles tendon, and **c** knees of the 6-year-old proband. **d** Sanger sequences of the *ABCG8* gene. A heterozygous nonsense variant, c.225G > A (p.Trp75*), was identified in the proband and her father (left panels, depicted by an arrow), whereas a heterozygous missense variant, c.1256_1257delTCinsAA (p.Ile419Lys), was observed in the proband, her mother, and her elder sister (right panels, depicted by arrows). Thus, these two variants were inherited in trans. **e** Homologs of the *ABCG8* gene at the Ile419 residue are well conserved across multiple species (shown in bold letters).

Considering these symptoms, we presumed that she had sitosterolemia and performed the genetic test. After obtaining written informed consent from the patient’s parents and approval from the local institutional review board, genomic DNA was extracted from the peripheral leukocytes of the patient and her family members. Sanger sequencing of 13 exons as well as the intron-exon boundaries of the *ABCG8* gene revealed a heterozygous nonsense variant in exon 3, NM_022437.3:c.225G > A (p.Trp75*), and two adjacent heterozygous missense variants in exon 9, NM_022437.3:c.1256T > A (p.Ile419Asn) and c.1257C > A (p.Ile419Ile), in the patient (Fig. 1d). The father of the patient had the variant c.225G > A (p.Trp75*), whereas the mother and the elder sister of the patient had the two adjacent variants c.1256T > A (p.Ile419Asn) and c.1257C > A (p.Ile419Ile) in a heterozygous state; this indicated that the patient had compound heterozygous variants in the *ABCG8* gene. No variants in the *ABCG5* gene were detected in the patient. These results confirmed the diagnosis of sitosterolemia. A low-plant-sterol diet, along with the administration of colestimide and ezetimibe, led to marked improvement in the patient and remedied the arthralgia, xanthomas, atherosclerotic plaque, and aortic valve regurgitation within 2 years. No worsening of the symptoms has been observed in 7 years of follow-up with this treatment regimen, except that the serum sitosterol level has remained elevated (approximately 100 µg/mL).

The nonsense variant c.225G > A (p.Trp75*) in the *ABCG8* gene, detected in the patient and her father, was not registered in population databases such as the Genome Aggregation Database (gnomAD, https://gnomad.broadinstitute.org/), Single Nucleotide Polymorphism Database (dbSNP, https://www.ncbi.nlm.nih.gov/snp/), Human Genetic Variation Database (HGVD, http://www.hgvd.genome.med.kyoto-u.ac.jp/) and ToMMo 4.7KJPN Allele Frequency Panel (https://jmorp.megabank.tohoku.ac.jp/202001/variants) or in disease databases, including the Human Gene Mutation Database (HGMD, https://portal.biobase-international.com/hgmd/pro/start.php) and ClinVar (https://www.ncbi.nlm.nih.gov/clinvar/). Located in exon 3 of the *ABCG8* gene, this variant results in a premature termination codon, and the resultant transcripts are presumably degraded by nonsense-mediated mRNA decay.

Two more variants in the *ABCG8* gene were detected in the patient, her mother, and her elder sister (c.1256T > A (p.Ile419Asn) and c.1257C > A (p.Ile419Ile) in exon 9). These were present in the same haplotype and were inherited together from the mother to her two daughters. Therefore, we interpreted these variants as a multiple-nucleotide variant (MNV) rather than as two independent single-nucleotide variants (SNVs). MNV is defined as a variant that possesses multiple substitutions within the same codon in the same haplotype^4,5^. In other words, in this situation, these variants should be regarded as a 2 bp deletion/insertion (indel), c.1256_1257delTCinsAA (p.Ile419Lys), rather than two adjacent but independent SNVs, namely, c.1256T > A (p.Ile419Asn) and c.1257C > A (p.Ile419Ile). This variant has also not been documented as a 2 bp indel in the previously mentioned population databases, except in gnomAD. However, the two adjacent SNVs, c.1256T > A (p.Ile419Asn) and c.1257C > A (p.Ile419Ile), in exon 9 are independently registered in the Japanese population databases HGVD and ToMMo 4.7KJPN at very low frequencies (Table 1). The attributes of these two SNVs were identical; i.e., the allele counts/allele frequencies of these SNVs were exactly the same in both databases. Furthermore, these two SNVs were individually registered in gnomAD as well and mostly detected in the East Asian population. Additionally, gnomAD states that these two SNVs were found in phase only in 16 East Asian individuals. Taking these results together, these two SNVs are assumed to form a 2 bp indel as an MNV, which is specifically observed in the East Asian population.

**Table 1:** Details of the multi-nucleotide variant (MNV) and corresponding single-nucleotide variants (SNVs) in the *ABCG8* gene.

Nucleotide	Amino acid	Population database				Disease database		*in silico* analysis			
		gnomAD^a,b^	dbSNP^c^	HGVD^a,d^	ToMMo 4.7KJPN^a,e^	HGMD^f^	ClinVar^g^	CADD score^h^	Polyphen-2^i^	SIFT^j^	MutationTaster^k^
<MNV detected in the patient>											
c.1256_1257delTCinsAA	p.Ile419Lys	0.000063 (16/251446)	—	—	—	Disease causing (hypercholesterolemia)	—	25.2	Probably damaging	Deleterious	Disease causing
<corresponding SNVs>											
c.1256T> A	p.Ile419Asn	0.000072 (18/251438)	rs201659189	0.0021 (5/1209)	0.0008 (8/9546)	—	—	26.6	Probably damaging	Deleterious	Disease causing
c.1257C > A	p.Ile419Ile	0.000063 (16/251446)	rs200818073	0.0021 (5/1209)	0.0008 (8/9546)	—	—	8.8	—	Tolerated	Disease causing

^a^Allele frequency and allele count are shown.

^b^Genome Aggregation Database; https://gnomad.broadinstitute.org/.

^c^Single Nucleotide Polymorphism Database; https://www.ncbi.nlm.nih.gov/snp/.

^d^Human Genetic Variation Database; http://www.hgvd.genome.med.kyoto-u.ac.jp/.

^e^ToMMo 4.7KJPN Allele Frequency Panel; https://jmorp.megabank.tohoku.ac.jp/202001/variants.

^f^Human Gene Mutation Database; https://portal.biobase-international.com/hgmd/pro/start.php.

^g^https://www.ncbi.nlm.nih.gov/clinvar/.

^h^https://cadd.gs.washington.edu/.

^i^http://genetics.bwh.harvard.edu/pph2/.

^j^https://sift.bii.a-star.edu.sg/.

^k^http://www.mutationtaster.org/.

Determining the pathogenicity of this MNV requires further investigation. For *in silico* analyses, this MNV showed a CADD score^6^ (https://cadd.gs.washington.edu/) of 25.2 and was predicted to be “Deleterious” with a score of 0.05 by SIFT^7^ (https://sift.bii.a-star.edu.sg/), “Probably damaging” with a score of 0.981 by PolyPhen-2^8^ (http://genetics.bwh.harvard.edu/pph2/), and “Disease causing” by MutationTaster^9^ (http://www.mutationtaster.org/) (Table 1). The isoleucine at codon 419 is well conserved across the species evaluated (Fig. 1e). According to the American College of Medical Genetics and Genomics/the Association for Molecular Pathology (ACMG/AMP) standards and guidelines for the interpretation of sequence variants^10^, this MNV satisfied the PM1 (Located in a mutational hot spot and/or critical and well-established functional domain without benign variation), PM2 (Absent from controls in the population databases), PM3 (For recessive disorders, detected in trans with a pathogenic variant), and PP3 (Multiple lines of computational evidence support a deleterious effect on the gene or gene product) criteria and thus was classified as “Likely pathogenic”. Furthermore, of particular interest is the report that one patient with familial hypercholesterolemia (FH) was found to carry the same MNV in a heterozygous state through a genetic screening of 96 Singaporean FH patients^11^. Following this report, this MNV was registered as a “Disease causing mutation” for hypercholesterolemia in HGMD (Table 1). Indeed, several heterozygous pathogenic variants in the *ABCG5* and *ABCG8* genes were identified with autosomal dominant segregation in FH families not carrying pathogenic variants in FH-related genes such as *LDLR*, *APOB*, and *PCSK9*^12–14^. However, hypercholesterolemia caused by heterozygous pathogenic variants in the *ABCG5* and *ABCG8* genes exhibits incomplete penetrance and variable expressivity^13,14^, as observed in our patient’s parents and sister. Of note, serum sitosterol levels in heterozygous *ABCG5* or *ABCG8* variant carriers are increased by at most 2-fold, which is modest compared to the at least 50-fold increases usually observed in patients with sitosterolemia caused by homozygous or compound heterozygous variants^14,15^. Taken together, these results indicate that the MNV c.1256_1257delTCinsAA (p.Ile419Lys), specifically observed in the East Asian population, may be causative not only for FH in a heterozygous state but also for sitosterolemia in a homozygous or compound heterozygous state.

In conclusion, we report a Japanese girl with sitosterolemia who harbors a novel nonsense SNV and an East Asian population-specific missense MNV in a compound heterozygous state. In sitosterolemia, prognosis can be improved by proper management, such as restriction of plant sterol intake and administration of a cholesterol absorption inhibitor^1^, as observed in our patient. Therefore, genetic testing of the *ABCG5* and *ABCG8* genes will play a crucial role in the accurate and early diagnosis of sitosterolemia. Further investigations and case studies are necessary to accumulate sufficient clinical evidence for genetic testing for sitosterolemia.

## Data Availability

The relevant data from this Data Report are hosted at the Human Genome Variation Database at 10.6084/m9.figshare.hgv.2897.

## References

[1] Yoo EG (2016). Sitosterolemia: a review and update of pathophysiology, clinical spectrum, diagnosis, and management. Ann. Pediatr. Endocrinol. Metab..

[2] Berge KE (2000). Accumulation of dietary cholesterol in sitosterolemia caused by mutations in adjacent ABC transporters. Science.

[3] Lu K (2001). Two genes that map to the STSL locus cause sitosterolemia: genomic structure and spectrum of mutations involving sterolin-1 and sterolin-2, encoded by ABCG5 and ABCG8, respectively. Am. J. Hum. Genet..

[4] Lek M (2016). Analysis of protein-coding genetic variation in 60,706 humans. Nature.

[5] Wang Q (2020). Landscape of multi-nucleotide variants in 125,748 human exomes and 15,708 genomes. Nat. Commun..

[6] Rentzsch P, Witten D, Cooper GM, Shendure J, Kircher M (2019). CADD: predicting the deleteriousness of variants throughout the human genome. Nucleic Acids Res..

[7] Vaser R, Adusumalli S, Leng SN, Sikic M, Ng PC (2016). SIFT missense predictions for genomes. Nat. Protoc..

[8] Adzhubei IA (2010). A method and server for predicting damaging missense mutations. Nat. Methods.

[9] Schwarz JM, Cooper DN, Schuelke M, Seelow D (2014). MutationTaster2: mutation prediction for the deep-sequencing age. Nat. Methods.

[10] Richards S (2015). Standards and guidelines for the interpretation of sequence variants: a joint consensus recommendation of the American College of Medical Genetics and Genomics and the Association for Molecular Pathology. Genet. Med..

[11] Pek SLT (2018). Spectrum of mutations in index patients with familial hypercholesterolemia in Singapore: single center study. Atherosclerosis.

[12] Lamiquiz-Moneo I (2017). ABCG5/G8 gene is associated with hypercholesterolemias without mutation in candidate genes and noncholesterol sterols. J. Clin. Lipidol..

[13] Tada H (2019). Rare and deleterious mutations in ABCG5/ABCG8 genes contribute to mimicking and worsening of familial hypercholesterolemia phenotype. Circ. J..

[14] Reeskamp LF (2020). ABCG5 and ABCG8 genetic variants in familial hypercholesterolemia. J. Clin. Lipidol..

[15] Tada H (2018). Oligogenic familial hypercholesterolemia, LDL cholesterol, and coronary artery disease. J. Clin. Lipidol..

